# Supplementary material for: Superconductivity from energy fluctuations in dilute quantum critical polar metals
Source: Nat Commun. 2022 Aug 6;13:4599. doi: 10.1038/s41467-022-32303-2 (PMC9357083; doi:10.1038/s41467-022-32303-2)
Supplement: Supplementary file 1 — Supplementary Information [file 41467_2022_32303_MOESM1_ESM.pdf]

Contents

1 Action of the Model **2**

2 Effects of Quantum Critical Fluctuations in the Normal State **2**

3 Interactions between Electrons in a Quantum Critical Polar Metal **3**

3.1 Screened Coulomb repulsion . . . 4

3.2 Electron-electron interaction from energy fluctuation exchange . . . 6

3.2.1 TO phonon contribution . . . 8

3.2.2 LO phonon contribution . . . 10

3.2.3 Cross-polarization contributions . . . 10

3.3 Expressions for 2D case . . . 11

3.3.1 Energy fluctuation exchange . . . 12

3.3.2 Cross-polarization term and the LO phonon contribution . . . 13

4 Superconductivity from Energy Fluctuations **14**

4.1 General relations in 3D . . . 14

4.2 Application to bulk doped $SrTiO_3$ . . . 15

4.3 2D layers . . . 16

1 Action of the Model

We consider a single band of conduction electrons in the presence of a soft polar phonon:

$$
\begin{aligned}
S &= S_e + S_{ph} + S_{En};\\
S_e &= \int d\tau d\mathbf{r} \psi^\dagger \left[-\partial_\tau - \frac{\nabla^2}{2m} - \mu \right] \psi;\\
S_{ph} &= \int d\tau d\mathbf{r} \frac{1}{2\varepsilon_0 \Omega_0^2} \left[[\partial_\tau \mathbf{P}(\mathbf{r},\tau)]^2 + [\omega_T(-i\nabla)\mathbf{P}(\mathbf{r},\tau)]^2 \right] + u \int d\tau d\mathbf{r} |\mathbf{P}(\mathbf{r},\tau)|^4;\\
S_{En} &= g \int d\tau d\mathbf{r} \psi^\dagger(\mathbf{r},\tau)\psi(\mathbf{r},\tau)|\mathbf{P}(\mathbf{r},\tau)|^2 \qquad (1)\\
S_{Coul} &= \frac{1}{2} \int d^d r d\tau \sum_{\alpha,\beta} \frac{\rho_{tot}(\mathbf{r},\tau)\rho_{tot}(\mathbf{r}',\tau)}{4\pi\varepsilon_0\varepsilon_1 |\mathbf{r}-\mathbf{r}'|},\\
\rho_{tot}(\mathbf{r}) &= \left[-\nabla \cdot \mathbf{P}(\mathbf{r},\tau) + e \sum_\alpha \psi_\alpha^\dagger(\mathbf{r},\tau)\psi_\alpha(\mathbf{r},\tau) \right],
\end{aligned}
$$

where $\omega_T(-i\nabla)$ is the transverse optic phonon dispersion (in momentum space, on has $(-i\nabla) \to \mathbf{q}$), ε_1 is the dielectric constant in the absence of the soft polar phonon.

2 Effects of Quantum Critical Fluctuations in the Normal State

First we consider the effects of the coupling of the conduction electrons to the energy fluctuations in the normal state. In particular, we can determine whether for weak coupling g the system can still remain in a Fermi liquid state at the QCP. For the purpose of this section we neglect the effects of the Coulomb interaction, which we assume to be screened in a metal and lead only to Fermi-liquid like corrections. The action in momentum space is:

$$
\begin{aligned}
S &= S_e + S_{ph} + S_{En};\\
S_e &= \int \frac{d\varepsilon d\mathbf{p}}{(2\pi)^{d+1}} \psi^\dagger \left[i\varepsilon - \frac{\mathbf{p}^2}{2m} + \mu \right] \psi;\\
S_{ph} &= \frac{1}{2} \int \frac{d\omega d\mathbf{q}}{(2\pi)^{d+1}} P_{\omega,\mathbf{q}} (\omega^2 + c^2 q^2) P_{-\omega,-\mathbf{q}} + u \left[\int \frac{d\omega d\mathbf{q}}{(2\pi)^{d+1}} \right]^3 |\mathbf{P}|^2 |\mathbf{P}|^2;\\
S_{En} &= g \int \frac{d\varepsilon d\mathbf{p}}{(2\pi)^{d+1}} \left[\int \frac{d\omega d\mathbf{q}}{(2\pi)^{d+1}} \right]^2 \psi^\dagger \psi |\mathbf{P}|^2
\end{aligned}
\tag{2}
$$

Assuming $z = 1$ one gets $[\psi] = -3/2$, $[P] = -(d+3)/2$

$$
[g] = -[2 + 2(d+1) - 3 - (d+3)] = 2 - d, \tag{3}
$$

and

$$
[u] = -[3(d+1) - 2(d+3)] = 3 - d. \tag{4}
$$

For the NFL physics the relevant exponent is the anomalous dimension of P:

$$
\langle P(\mathbf{r}) P(\mathbf{r}') \rangle \sim \frac{1}{|\mathbf{r} - \mathbf{r}'|^{D-2+\eta}} \to \langle P(\mathbf{q}) P(-\mathbf{q}) \rangle \sim \frac{1}{|\mathbf{q}|^{2-\eta}}, \tag{5}
$$

such that $[P]' = -(d + 3 - \eta)/2$ and $g' = 2 - d - \eta$. As $\eta > 0$ for local QFTs [1], g becomes irrelevant.

However, for bosons the effects of g can still be relevant [2] for two and three dimensions, as in second order it induces a quartic bosonic term. On the other hand, the long-range Coulomb interaction, if the fermions are charged, makes this term irrelevant. This occurs because the RPA screening of the density-density response function implies $\Pi_{RPA}(\mathbf{q} \to 0, \omega = 0) \sim q^{d-1}$.

3 Interactions between Electrons in a Quantum Critical Polar Metal

We can now study the effects of the coupling to TO phonon energy S_{En} (1) perturbatively. In particular, we study the resulting electron-electron interaction. In what follows, we will ignore the effects of the quartic phonon-phonon interaction u, Eq. (1) (all equations and figure refer to SM unless otherwise stated), assuming the relevant energy/momenta to be larger than the critical scale, set by the value of u.

In lowest order in g the effective interaction is given by the Feynman diagrams in Fig. 1 (a). For the Coulomb repulsion (blue dashed line, Fig. 1 (b)) we take the electronic screening in the RPA approximation (to be justified below); the screening by the polar mode includes the lowest-order (Hartree) correction from the energy interactions. At the same time, the polar phonon propagator (red wavy line, Fig. 1 (c)) receives corrections from the finite electron density due to energy interactions (first self-energy diagram) and Coulomb repulsion, screened by conduction electrons (next self-energy diagrams).

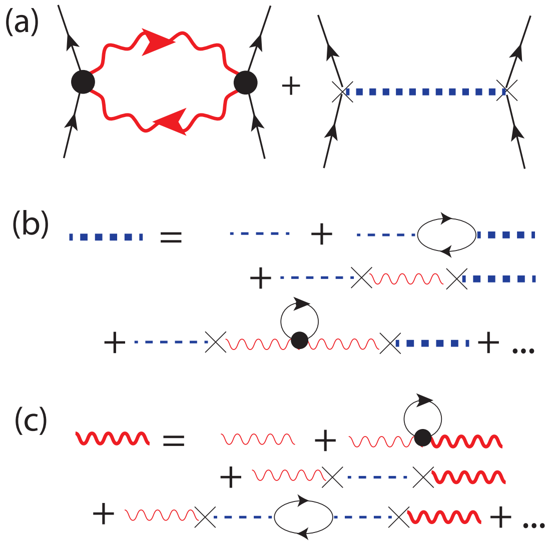

Figure 1: (a) Effective interaction between electrons (b) Renormalization of the phonon propagator (red wavy line) and the Coulomb (blue dashed line) interaction. Crosses are vertices for the Coulomb interaction S_{Coul}, while full circles - for the coupling to phonon energy S_{En} (see (1)).

3.1 Screened Coulomb repulsion

We start with the Coulomb interaction which is screened by both polar phonons and conduction electrons. We can now write the fully screened Coulomb repulsion in a doped polar metal in the following form (in Matsubara frequencies):

$$V_C(\mathbf{q},\omega_n) = \frac{e^2/\varepsilon_0}{\left(\varepsilon_1 + \frac{\Omega_0^2}{\omega_n^2+\omega_T^2(\mathbf{q})}\right) q^2 - e^2\Pi_{el}(\mathbf{q},\omega_n)/\varepsilon_0}, \tag{6}$$

where $\Pi_{el}(\mathbf{q}, \omega_n)$ is the polarization operator of the conduction electrons.

In the absence of the conduction electrons, an empirical relation for the low-temperature dielectric constant $\varepsilon(T) = \frac{\Omega_0^2}{\omega_T^2(\mathbf{q}=0)}$ [3] holds. It also follows that

$$\Omega_0^2 = \varepsilon_1(\omega_L^2(\mathbf{q}) - \omega_T^2(\mathbf{q})), \tag{7}$$

and thus Ω_0^2 is generally expected to be larger than $\omega_L^2 - \omega_T^2$.

In the presence of a finite electron density, one has to include RPA corrections as well as a Hartree phonon self-energy from energy interactions. The latter results in a electron-density-dependent shift of the transverse phonon energy:

$$\omega_T^2(n_e, \mathbf{q}) = \omega_T^2(\mathbf{q}) + 2g\varepsilon_0 n_e \Omega_0^2. \tag{8}$$

For $qv_F \gg \omega_n$ one can substitute $-(e^2/\varepsilon_0)\Pi_{el}(\mathbf{q}, \omega_n)$ for κ_D^2 — square of the (unscreened) inverse Debye length. This leads to the well-known Bardeen-Pines interaction:

$$V_C(\mathbf{q}, \omega_n) \approx \frac{e^2/\varepsilon_0}{\varepsilon_1 q^2 + \kappa_D^2} \left(1 - \frac{\frac{\Omega_0^2 q^2}{\varepsilon_1 q^2 + \kappa_D^2}}{\omega_n^2 + \frac{\Omega_0^2 q^2}{\varepsilon_1 q^2 + \kappa_D^2}} \right), \tag{9}$$

where the attractive part of the interaction is retarded. However, as the retardation effects occur on the scale of the order ω_L, the frequency dependence of the gap can not lead to a finite T_c in the regime $\omega_T \gg E_F$.

On the other hand, for $qv_F \ll \omega_n$, $-4\pi e^2 \Pi_{el}(\mathbf{q}, \omega_n) \approx \frac{\omega_p^2}{\omega^2} q^2$, leading to a plasmon pole at $\omega_p/\varepsilon_0 \ll \omega_p$. In what follows we will neglect this contribution to pairing, since it is suppressed by a factor $\varepsilon^{-1} \ll 1$ [4].

Thus, to estimate T_c we use the form of the interaction for $\mathbf{q} = \mathbf{k} - \mathbf{k}'$, where both are close to the Fermi surface ($|k - k_F| \ll k_F$), assume $qv_F, \omega_T \gg \omega_n$, and take the static $q = 0$ value of the polarization operator. We also assume $k_F \ll q_D$ for low densities allowing us to expand the TO phonon dispersion for low q $\omega_T^2(n_e, \mathbf{q}) \approx \omega_T^2(n_e) + c_s^2 \vec{q}^2$:

$$V_C^{eff}(k, k', \omega_n) \approx \left\langle \frac{e^2/\varepsilon_0}{\left(\varepsilon_1 + \frac{\Omega_0^2}{\omega_T^2(n_e) + c_s^2 |\mathbf{k} - \mathbf{k}'|^2} \right) |\mathbf{k} - \mathbf{k}'|^2 + \kappa_D^2} \right\rangle_\theta, \tag{10}$$

where θ is the angle between $\mathbf{k}$ and $\mathbf{k}'$. To simplify the expression above, let us consider the orders of magnitude of the various terms. Close to the polar QCP, we can expect ε_1 to be negligible compared to the "second term" of order $\varepsilon \gg 1$. Comparing the two remaining terms in the denominator we find that the first is of

the order $\Omega_0^2 k_F^2/(\omega_T^2 + c_s^2 k_F^2)$ (the corrections to $\omega_T^2(n_e)$ are $\sim n_e \sim k_F^3$ and can be neglected for low densities), while $\kappa_D^2 \sim k_F/a_B$, where a_B - is the Bohr radius. At the QCP $\omega_T^2 = 0$ one finds that the first one is always dominant at low densities. On the other hand, in the non-critical regime $\omega_T^2 \gtrsim c_s^2 k_F^2$, the ratio of the κ_D^2 term is smaller by $1/(\varepsilon k_F a_B)$, which we assume to be much smaller then 1 for the Mott criterion for metallicity to be satisfied. (Indeed it is much larger for the case of $SrTiO_3$ [5]). Consequently, we neglect κ_D in the denominator in (10). The effective renormalized Coulomb repulsion is then

$$V_C^{eff}(k,k',\omega_n) \approx \left\langle \frac{(e^2/\varepsilon_0)(\omega_T^2 + c_s^2|\mathbf{k}-\mathbf{k}'|^2)}{\Omega_0^2|\mathbf{k}-\mathbf{k}'|^2} \right\rangle_\theta = \frac{e^2\omega_T^2 \log\left|\frac{|\mathbf{k}|+|\mathbf{k}'|}{|\mathbf{k}|-|\mathbf{k}'|}\right|}{2\varepsilon_0\Omega_0^2|\mathbf{k}||\mathbf{k}'|} + \frac{e^2c_s^2}{\varepsilon_0\Omega_0^2}. \tag{11}$$

One can further simplify the expression above by restricting the momenta in (11) to the vicinity of the Fermi surface and performing an average:

$$\frac{\log\left|\frac{|\mathbf{k}|+|\mathbf{k}'|}{|\mathbf{k}|-|\mathbf{k}'|}\right|}{|\mathbf{k}||\mathbf{k}'|} \approx \frac{1}{2k_F k_F^2}\int_{-k_F}^{k_F} dk \log\left|\frac{2k_F}{k}\right| = \frac{1+\log 2}{k_F^2}. \tag{12}$$

If the average is performed for $\mathbf{k}, \mathbf{k}'$ being within the Fermi sphere of the radius k_F, the result is $\langle |\mathbf{k}-\mathbf{k}'|^{-2}\rangle = \frac{1}{(k_F^2/3)^2}\int_0^{k_F} dk dk' kk' \log\left|\frac{|\mathbf{k}|+|\mathbf{k}'|}{|\mathbf{k}|-|\mathbf{k}'|}\right| = \frac{9}{2k_F^2}$, i.e. only the prefactor is somewhat different.

The resulting dimensionless coupling constant $\nu_0 V_{eff}$, where $\nu_0 = \frac{m^* k_F}{2\pi^2\hbar^2}$ is the density of states can be estimated:

$$V_C^{eff}(k,k',\omega_n) \approx \frac{(e^2/\varepsilon_0)\omega_T^2(1+\log 2)}{2\Omega_0^2 k_F^2} + \frac{e^2c_s^2}{\varepsilon_0\Omega_0^2}. \tag{13}$$

3.2 Electron-electron interaction from energy fluctuation exchange

Let us now consider the effective interaction induced by the energy fluctuation exchange term (Fig. 2):

$$\begin{aligned} \Delta S = -\frac{1}{2}g^2 \int d\mathbf{r}d\mathbf{r}' V_{En}(\mathbf{r}-\mathbf{r}',\tau-\tau')\psi_\sigma^\dagger(\mathbf{r},\tau)\psi_\sigma(\mathbf{r},\tau)\psi_\sigma^\dagger(\mathbf{r}',\tau')\psi_\sigma(\mathbf{r}',\tau'); \\ V_{En}(i\omega_n,\mathbf{q}) = 2T\sum_{\omega_n',\mathbf{q}'} \mathrm{Tr}[D_{\alpha\beta}(\omega_n'+\omega_n,\mathbf{q}'+\mathbf{q})D_{\beta\alpha}(\omega_n',\mathbf{q}')]. \end{aligned} \tag{14}$$

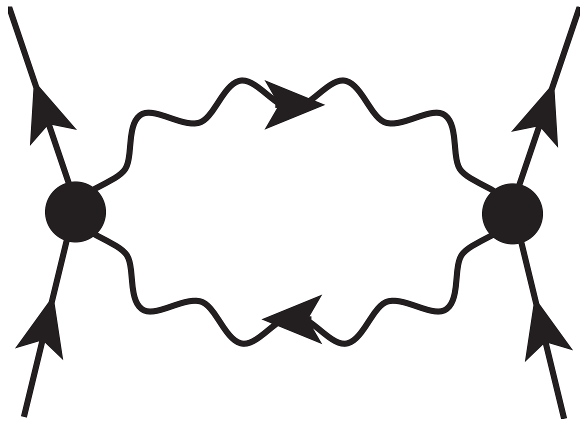

Figure 2: Induced electron-electron interaction from the energy fluctuation exchange (14)

For the phonon propagators in (14), we need to include the effects of Coulomb interaction and phonon frequency renormalization by the electrons due to the energy coupling S_{En} (1) (Fig. 1 (c)). The bare polar phonon propagator is

$$D^{(0)}_{\alpha\beta}(i\omega_n, \mathbf{q}) = \frac{\varepsilon_0 \Omega_0^2}{\omega_n^2 + \omega_T^2 + c_s^2 \mathbf{q}^2} \tag{15}$$

In this subsection we will follow the notation (15). The renormalization of the TO phonon frequency (8) does not change the form of the propagator, while the Coulomb repulsion (screened by the conduction electrons) does:

$$\varepsilon_0 D^{-1}_{\alpha\beta}(i\omega_n, \mathbf{q}) = \frac{\omega_n^2 + c_s^2 \mathbf{q}^2 + \omega_T^2}{\Omega_0^2}\delta_{\alpha\beta} + \frac{q^\alpha q^\beta}{\varepsilon_1 q^2 + \kappa_D^2}, \tag{16}$$

where we have used the RPA expression (see above) for the electronic screening in the limit $v_F q \gg \omega_n$. The propagator is then obtained using a mathematical identity:

$$(A_\alpha \delta_{\alpha\beta} + B q_\alpha q_\beta)^{-1} = \frac{\delta_{\alpha\beta}}{A_\alpha} - \frac{B q_\alpha q_\beta}{A_\alpha A_\beta \left(1 + B \sum_\gamma \frac{q_\gamma^2}{A_\gamma}\right)};$$

$$\varepsilon_0^{-1} D_{\alpha\beta}(\mathbf{q}, \omega_n) = \frac{\Omega_0^2 \delta_{\alpha\beta}}{\omega_n^2 + \omega_T^2 + c_s^2 \mathbf{q}^2} - \frac{\Omega_0^2}{(\omega_n^2 + \omega_T^2 + c_s^2 \mathbf{q}^2)} \frac{\frac{q^\alpha q^\beta \Omega_0^2}{\varepsilon_1 q^2 + \kappa_D^2}}{\left(\omega_n^2 + \omega_T^2 + c_s^2 \mathbf{q}^2 + \frac{\mathbf{q}^2 \Omega_0^2}{\varepsilon_1 q^2 + \kappa_D^2}\right)}, \tag{17}$$

where in the absence of conduction electrons one obtains the expected LO-TO splitting.

We can now evaluate the trace in (14) using simplified notation

$$
\begin{aligned}
D(i\omega_n^{1(2)}, \mathbf{q}^{1(2)}) &= \frac{\delta_{\alpha\beta}}{A^{1(2)}} - \frac{B^{1(2)} q_\alpha q_\beta}{A^{1(2)}(A^{1(2)} + B^{1(2)} q^2)}, \\
A^{1(2)} &= \frac{\omega_n^2 + \omega_T^2 + c_s^2 \mathbf{q}_{1(2)}^2}{\varepsilon_0 \Omega_0^2}, \\
B^{1(2)} &= \frac{q_{1(2)}^\alpha q_{1(2)}^\beta}{\varepsilon_0(\varepsilon_1 q_{1(2)}^2 + \kappa_D^2)}.
\end{aligned}
\tag{18}
$$

One obtains

$$
\begin{aligned}
\mathrm{Tr}[\hat{D}(i\omega_n^1, \mathbf{q}^1)\hat{D}(i\omega_n^2, \mathbf{q}^2)] &= \frac{3}{A_1 A_2} - \frac{B_1 \mathbf{q}_1^2}{A_1 A_2 (A_1 + B_1 \mathbf{q}_1^2)} - \frac{B_2 \mathbf{q}_2^2}{A_1 A_2 (A_2 + B_2 \mathbf{q}_2^2)} \\
&+ \frac{B_1 B_2 (\mathbf{q}_1 \cdot \mathbf{q}_2)^2}{A_1 A_2 (A_1 + B_1 \mathbf{q}_1^2)(A_2 + B_2 \mathbf{q}_2^2)} = \\
&= \frac{1}{A_1 A_2} + \frac{1}{A_2 (A_1 + B_1 \mathbf{q}_1^2)} + \frac{1}{A_1 (A_2 + B_2 \mathbf{q}_2^2)} \\
&+ \frac{B_1 B_2 [(\mathbf{q}_1 \cdot \mathbf{q}_2)^2 \pm \mathbf{q}_1^2 \mathbf{q}_2^2]}{A_1 A_2 (A_1 + B_1 \mathbf{q}_1^2)(A_2 + B_2 \mathbf{q}_2^2)} = \\
= \frac{2}{A_1 A_2} &+ \frac{B_1 B_2 [(\mathbf{q}_1 \cdot \mathbf{q}_2)^2 - \mathbf{q}_1^2 \mathbf{q}_2^2]}{A_1 A_2 (A_1 + B_1 \mathbf{q}_1^2)(A_2 + B_2 \mathbf{q}_2^2)} + \frac{1}{(A_1 + B_1 \mathbf{q}_1^2)(A_2 + B_2 \mathbf{q}_2^2)},
\end{aligned}
\tag{19}
$$

The first term in the above is readily recognized to be the contribution of two transverse optical phonons, as the Coulomb renormalization B is completely absent from this term. In the same way, third term is the contribution of the LO phonon. The second one is a cross-term, that can be seen to vanish at $\mathbf{q}_1 = \mathbf{q}_2$ — for zero incoming momenta in the phonon loop, the momenta in the propagators are the same and LO and TO polarization vectors are orthogonal to one another, i.e. $(e_T^{1,2}(\mathbf{q}) \cdot e_{LO}(\mathbf{q})) = 0$. However if the momenta of the two propagators are not the same a cross term may occur as $(e_T^{1,2}(\mathbf{q}_1) \cdot e_{LO}(\mathbf{q}_2)) \neq 0$.

3.2.1 TO phonon contribution

Now, let us move on to the calculation of the frequency/momentum integrals in the first (TO-related) term of (19). Assuming weak-coupling $T_c \ll \omega_T, c_s k_F$ we may take the limit $T \to 0$ in the Matsubara sums, transforming them into integrals.

Furthermore, we will use hyperspherical coordinates and use an overall cutoff for $\sqrt{\omega^2 + c_s^2 q^2}$, rather then only q - this does not affect the leading logarithmic contribution (see below for the discussion of corrections). The integrals can be calculated using Feynman's trick:

$$XY = \int_0^1 \frac{du}{(uX + (1-u)Y)^2}. \tag{20}$$

leading to

$$\begin{aligned}
&4\Omega_0^4 \varepsilon_0^2 \times | \int \frac{d\omega'}{2\pi} \int_{q'<q_D} \frac{d\mathbf{q}'}{(2\pi)^3} \frac{1}{(\omega'+\omega)^2 + c_s^2(\mathbf{q}'+\mathbf{q})^2 + \omega_T^2} \frac{1}{\omega^2 + c_s^2 q^2 + \omega_T^2} \approx \\
&\approx \frac{1}{(2\pi)^4 c_s^3} \int_{x' \lesssim \Omega_T} d^4x' \frac{1}{\sum_{i=0,1,2,3}(x' - \omega_q)_i^2 + \omega_T^2} \frac{1}{x'^2 + \omega_T^2} = \\
&\frac{1}{(2\pi)^4 c_s^3} \int_{x' \lesssim \Omega_T} d^4x' \int_0^1 du \frac{1}{\left(\sum_{i=0,1,2,3}(x' - (1-u)\omega_q)_i^2 + \omega_T^2 + u(1-u)\omega_q^2\right)^2} \approx \\
&\approx \frac{1}{(2\pi)^4 c_s^3} \int_0^1 du \pi^2 \left(\log\left[\frac{\Omega_T^2}{u(1-u)\omega_q^2 + \omega_T^2}\right] - 1 \right) = \\
&= \frac{1}{8\pi^2 c_s^3} \left(\log\left[\frac{\Omega_T}{\omega_T}\right] + \frac{1}{2} - \frac{\sqrt{4\omega_T^2 + \omega_q^2}}{2\omega_q} \log\left[\frac{\sqrt{4\omega_T^2 + \omega_q^2} + \omega_q}{\sqrt{4\omega_T^2 + \omega_q^2} - \omega_q}\right] \right) \\
&\approx_{\omega_q, \omega_T \ll \Omega_T} \frac{1}{8\pi^2 c_s^3} \left(\frac{1}{2} + \log\frac{\Omega_T}{\omega_T} - f(\omega_q/\omega_T) \right),
\end{aligned} \tag{21}$$

where $\omega_q^i = (c_s\mathbf{q}, \omega)$; $\omega_q = \sqrt{c_s^2 q^2 + \omega^2}$ and $f(x)$ is given by:

$$f(x) = \frac{\sqrt{4+x^2}}{2x} \log\left[\frac{\sqrt{4+x^2} + x}{\sqrt{4+x^2} - x}\right] \tag{22}$$

Importantly, in the quantum critical regime $\omega_T \to 0$ (also corresponding to the case $c_s k_F \gg \omega_T$) one has $f(\omega_q/\omega_T) \approx \log\frac{\omega_q}{\omega_T}$, i.e. the lower cutoff of the logarithm is now given by ω_q instead of ω_T. In the opposite limit $\omega_q \ll \omega_T$, corresponding to low doping, $f(\omega_q/\omega_T) \approx 1$.

Let us now discuss the corrections to the above result due to the use of a combined energy-momentum cutoff instead of a momentum cutoff only. In particular, one has (here we take the incoming frequency/momentum as well as ω_T to be zero, as the

integrals are the same at low energies):

$$\begin{aligned}\int_{-\infty}^{\infty} d\omega \int_0^{q_D} q^2 dq \frac{1}{(\omega^2+\bar{c}_s^2 q^2)^2} - \int_{\omega^2+\bar{c}_s^2 q^2<\Omega_T^2;\, q>0} d\omega q^2 dq \frac{1}{(\omega^2+\bar{c}_s^2 q^2)^2} = \\ = \int_0^{\Omega_T} x^2 dx \frac{\pi}{2x^3} - \int_0^{\Omega_T} x^3 dx \frac{\pi}{2x^4} = 0.\end{aligned} \tag{23}$$

Thus, the corrections have to arise only from the low-energy region, where they are parametrically small, of the order $\omega_T^2, c_s^2 q^2, \omega^2/\Omega_T^2 \ll 1$.

3.2.2 LO phonon contribution

Here we calculate of the frequency/momentum integrals in the third (LO) term of (19).

$$\int \frac{d\omega}{2\pi} \int_{q<q_D} \frac{d\mathbf{q}}{(2\pi)^3} \frac{1}{\left[(\omega'+\omega)^2 + c_s^2(\mathbf{q}+\mathbf{q}')^2 + \frac{\Omega_0^2(\mathbf{q}+\mathbf{q}')^2}{\varepsilon_1(\mathbf{q}+\mathbf{q}')^2+\kappa_D^2}\right]\left[\omega^2 + c_s^2\mathbf{q}^2 + \omega_T^2 + \frac{\Omega_0^2\mathbf{q}^2}{\varepsilon_1\mathbf{q}^2+\kappa_D^2}\right]}. \tag{24}$$

Assuming in the low-density limit that $4\varepsilon k_F^2 \ll \kappa_D^2$ one can divide the integration in two regions: for $q \ll \kappa_D/\sqrt{\varepsilon_1}$ one has

$$\begin{aligned}\int \frac{d\omega}{2\pi} \int_{q<\kappa_D} \frac{d\mathbf{q}}{(2\pi)^3} \frac{1}{\left[(\omega'+\omega)^2 + \left(c_s^2 + \frac{\Omega_0^2}{\kappa_D^2}\right)(\mathbf{q}+\mathbf{q}')^2\right]\left[\omega^2 + \left(c_s^2 + \frac{\Omega_0^2}{\kappa_D^2}\right)\mathbf{q}^2 + \omega_T^2\right]} = \\ = \frac{1}{8\pi^2\left(c_s^2+\frac{\Omega_0^2}{\kappa_D^2}\right)^{3/2}} \left(\log\left[\frac{\sqrt{c_s^2 + \frac{\Omega_0^2}{\kappa_D^2}}\frac{\kappa_D}{\sqrt{\varepsilon_1}}}{\omega_T}\right] + \frac{1}{2} - \frac{\sqrt{4\omega_T^2+\omega_q^2}}{2\omega_q} \log\left[\frac{\sqrt{4\omega_T^2+\omega_q^2}+\omega_q}{\sqrt{4\omega_T^2+\omega_q^2}-\omega_q}\right]\right).\end{aligned} \tag{25}$$

One observes that while the argument of the logarithm is larger than Ω_T and is of the order ω_{LO}, the prefactor is smaller by a factor $\left(1+\frac{\Omega_0^2}{c_s^2\kappa_D^2}\right)^{-3/2}$.

3.2.3 Cross-polarization contributions

Finally, we account for the second term in (19) , which arises due to the momentum dependence of the LO/TO eigenvectors, and in particular for the fact that $(\mathbf{e}_T^{1,2}(\mathbf{q}_1)\cdot \mathbf{e}_{LO}(\mathbf{q}_2)) \neq 0$ for $\mathbf{q}_1 \neq \mathbf{q}_2$.

At low densities, such that the frequency exchanged ω is much smaller than the corresponding momentum scale: $E_F \ll c_s q \sim c_s k_F$, one can neglect the frequency in the two-phonon loop. As will be shown below, the momentum integral in the cross-term converges rapidly at $q \gg 2k_F$; consequently one can use $c_s^2 \ll \frac{\Omega_0^2}{\varepsilon_1 q^2 + \kappa_D^2}$ for such low momenta. Thus, one gets for the cross-term in the quantum critical regime $c_s k_F \gg \omega_T$:

$$\int \frac{d\omega}{2\pi} \int_{q<q_D} \frac{d\mathbf{q}}{(2\pi)^3} \frac{B_1 B_2 [(\mathbf{q}_1 \cdot \mathbf{q}_2)^2 - \mathbf{q}_1^2 \mathbf{q}_2^2]}{A_1 A_2 (A_1 + B_1 \mathbf{q}_1^2)(A_2 + B_2 \mathbf{q}_2^2)} \approx$$
$$\approx - \int \frac{d\omega}{2\pi} \int_{q<q_D} \frac{d\mathbf{q}}{(2\pi)^3} \frac{B(\mathbf{q_1}) B(\mathbf{q_2}) q^2 \delta^2 (\cos^2\theta - 1)}{(\omega^2 + c_s^2(\mathbf{q}+\delta/2)^2)(\omega^2 + c_s^2(\mathbf{q}-\delta/2)^2)(\omega^2 + B(\mathbf{q_1})\mathbf{q}_1^2)(\omega^2 + B(\mathbf{q_2})\mathbf{q}_2^2)} \approx_{\omega \sim c_s \delta}$$
$$\approx - \int \frac{d\omega}{2\pi} \int_{q<q_D} \frac{d\mathbf{q}}{(2\pi)^3} \frac{q^2\delta^2(\cos^2\theta - 1)/((q^2+\delta^2/4)^2 - q^2\delta^2\cos^2\theta)}{(\omega^2 + c_s^2(\mathbf{q}+\delta/2)^2)(\omega^2 + c_s^2(\mathbf{q}-\delta/2)^2)} =$$
$$= - \int_{q<q_D} \frac{d\mathbf{q}}{2(2\pi c_s)^3} \frac{q^2\delta^2(\cos^2\theta - 1)}{((q^2+\delta^2/4)^2 - q^2\delta^2\cos^2\theta)^{3/2}(\sqrt{q^2+\delta^2/4 - q\delta\cos\theta} + \sqrt{q^2+\delta^2/4+q\delta\cos\theta})}$$
$$\approx \int_0^\infty \frac{dx d\cos\theta}{8\pi^2 c_s^3} \frac{4x^4(\cos^2\theta - 1)}{((x^2+1)^2 - 4x^2\cos^2\theta)^{3/2}(\sqrt{x^2+1-2x\cos\theta} + \sqrt{x^2+1+2x\cos\theta})} \approx$$
$$\approx -\frac{0.81}{8\pi^2 c_s^3}. \tag{26}$$

3.3 Expressions for 2D case

We now deduce the energy-fluctuation coupling in 2D along the same lines. To make better connection with the 3D case, we consider the 2D system as a layer of the bulk material with thickness l_0. The electronic and phononic eigenfunctions are assumed to be in the lowest eigenstate along z direction such that $\Psi(\mathbf{r},\tau) \to \Psi(\mathbf{r}_{2D},\tau) d_\Psi(z)$; $\to \mathbf{P}(\mathbf{r},\tau) \to \mathbf{P}(\mathbf{r}_{2D},\tau) d_P(z)$, where the functions $d_{\Psi,P}(z)$ are approximately equal to $1/\sqrt{l_0}$ within the layer and zero outside.

Introducing these definitions into the interaction terms one gets:

$$g \int d\tau d\mathbf{r} \Psi^\dagger(\mathbf{r},\tau)\Psi(\mathbf{r},\tau)|\mathbf{P}|^2(\mathbf{r},\tau) \to \frac{g}{l_0} \int d\tau d\mathbf{r}_{2D} \Psi^\dagger(\mathbf{r}_{2D},\tau)\Psi(\mathbf{r}_{2D},\tau)|\mathbf{P}|^2(\mathbf{r}_{2D},\tau)$$
$$\rho_{tot}(\mathbf{r}) \to \left[e\Psi_\alpha^\dagger(\mathbf{r},\tau)\Psi_\alpha(\mathbf{r},\tau) - \sqrt{l_0}\nabla\cdot\mathbf{P}(\mathbf{r},\tau)\right]\delta(z),$$
$$V_{Coul}(\mathbf{q}) = \frac{1}{\varepsilon_0\varepsilon_1 q^2} \to \frac{1/(2\varepsilon_0)}{q + \frac{\varepsilon_1 l_0}{2} q^2}$$

where the expression for the dielectric screening for the thin film limit in vacuum [6, 7] has been used. Below we use the vector notation for the 2D vectors, omitting the "2D" index. To calculate the polar phonon propagator in a polar metal, we include RPA screening if the Coulomb repulsion by conduction electrons:

$$V_{Coul}^{cond}(\mathbf{q}) = \frac{e^2/(2\varepsilon_0)}{q + \frac{\varepsilon_1 l_0}{2} q^2 + \frac{1}{2\varepsilon_0} e^2 \nu \left(\equiv \frac{m^*}{\pi \hbar^2}\right)} \approx_{q \sim 2k_F \ll a_0^{-1}} \pi \frac{\hbar^2}{m^*}, \tag{27}$$

where the expression for 2D density of states is used; as $\frac{\hbar^2}{m^* e^2} \equiv a_B^* \sim a_B$ one can neglect the other terms in the denominator.

The polar phonon propagator (including the corrections due to Coulomb interactions, Fig. 1 (c)) takes then the form:

$$\varepsilon_0^{-1} D_{\alpha\beta}^{2D}(\mathbf{q}, \omega_n) = \frac{\Omega_0^2 \delta_{\alpha\beta}}{\omega_n^2 + \omega_T^2 + c_s^2 \mathbf{q}^2} - \frac{\Omega_0^2}{(\omega_n^2 + \omega_T^2 + c_s^2 \mathbf{q}^2)} \frac{q^\alpha q^\beta \Omega_0^2 l_0 a_B^*/2}{(\omega_n^2 + \omega_T^2 + c_s^2 \mathbf{q}^2 + \Omega_0^2 l_0 a_B^* \mathbf{q}^2/2)}. \tag{28}$$

Strictly speaking, for a thin film the out-of-plane and in-plane orientations become different, and a splitting is expected. However, in STO (where even the bulk is actually tetragonal and not cubic at low temperatures), this splitting is quite small (see below). Additionally, unlike 3D, in the absence of conduction electrons the LO-TO splitting would still vanish at $\mathbf{q} = 0$ [8].

Finally, the fully renormalized Coulomb interaction (Fig. 1 (b)) is:

$$V_{Coul}^{full}(\mathbf{q}) \approx_{q \sim 2k_F \ll a_0^{-1}} \frac{e^2 \varepsilon_0^{-1}}{\frac{\Omega_0^2 l_0 \mathbf{q}^2}{\omega_n^2 + \omega_T^2 + c^2 \mathbf{q}^2} + 4(a_B^*)^{-1}}. \tag{29}$$

3.3.1 Energy fluctuation exchange

The attraction due to the exchange of energy fluctuations (here the expression for the case of only one TO phonon present is given; if the out-of-plane mode is sufficiently soft, a factor of 2 should be added):

$$
\begin{aligned}
2\varepsilon_0^2\Omega_0^4| \frac{1}{(2\pi)^3 c_s^2}\int_{x\lesssim\Omega_T} d^3x \int_0^1 du \frac{1}{\left(\sum_{i=0,1,2}(x-(1-u)\omega_q)_i^2+\omega_T^2+u(1-u)\omega_q^2\right)^2} \approx \\
\approx \frac{\pi^2}{(2\pi)^3 c_s^2}\int_0^1 \frac{du}{\sqrt{u(1-u)\omega_q^2+\omega_T^2}} = \\
= \frac{1}{8\pi c_s^2\omega_T}\frac{\arctan\frac{\omega_q}{2\omega_T}}{\frac{\omega_q}{2\omega_T}}.
\end{aligned}
\tag{30}
$$

For the case of $c_s k_F \gtrsim \omega_T$ an angular average of the last term is performed as follows:

$$
\begin{aligned}
\left\langle \frac{\arctan\frac{\omega_q}{2\omega_T}}{\frac{\omega_q}{2\omega_T}} \right\rangle = \frac{1}{2\pi}\int_0^{2\pi} \frac{\arctan\frac{c_s k_F \sin\frac{\varphi}{2}}{\omega_T}}{\frac{c_s k_F \sin\frac{\varphi}{2}}{\omega_T}} d\varphi \approx 1(\omega_T \gg c_s k_F), \\
= (\omega_T \ll c_s k_F)\frac{\omega_T}{2c_s}\left\langle \int_0^{2\pi} d\varphi \frac{1}{\sqrt{4kk'\sin^2\frac{\varphi}{2}+(k-k')^2}} \right\rangle_{k-k'} = \\
= \frac{2\omega_T}{c_s}\int_{-k_F}^{k_F}\frac{dk}{2k_F}\frac{K[-4k_F^2/k^2]}{|k|} \approx \frac{3.06\omega_T}{c_s k_F}.
\end{aligned}
$$

3.3.2 Cross-polarization term and the LO phonon contribution

For the cross polarization term, one gets assuming $\Omega_0^2 l_0 a_B^*/2 \gg c_s^2$ in the quantum critical regime ($\omega_T \ll c_s k_F$):

$$
\begin{aligned}
2\varepsilon_0^2\Omega_0^4\times| - \int\frac{d\omega'}{2\pi}\int_{q'<q_D}\frac{d\mathbf{q}'}{(2\pi)^2}\frac{q'^2q^2(\cos^2\theta-1)/((q'^2+q^2/4)^2-q'^2q^2\cos^2\theta)}{(\omega'^2+c_s^2(\mathbf{q}'+\mathbf{q}/2)^2)(\omega'^2+c_s^2(\mathbf{q}'-\mathbf{q}/2)^2)} = \\
= -\int_{q'<q_D}\frac{d\mathbf{q}'}{2(2\pi)^2c_s^3}\frac{q'^2q^2(\cos^2\theta-1)}{((q'^2+q^2/4)^2-q'^2q^2\cos^2\theta)^{3/2}(\sqrt{q'^2+q^2/4-q'q\cos\theta}+\sqrt{q'^2+q^2/4+q'q\cos\theta})} \\
\approx \int_0^\infty \frac{dxd\theta}{4\pi^2 q c_s^3}\frac{4x^3(\cos^2\theta-1)}{((x^2+1)^2-4x^2\cos^2\theta)^{3/2}(\sqrt{x^2+1-2x\cos\theta}+\sqrt{x^2+1+2x\cos\theta})} \approx \\
\approx -\frac{2.09}{4\pi^2c_s^3|\mathbf{k}-\mathbf{k}'|}.
\end{aligned}
\tag{31}
$$

The term is of the same magnitude as the TO-exchange term. The LO term, on the other hand, is obtained from the TO contribution by substituting $c_s \rightarrow$

$\sqrt{c_s^2 + \Omega_0^2 l_0 a_B^*/2}$ and is thus much smaller then the TO term. Neglecting it, the total interaction is given by:

$$2\varepsilon_0^2\Omega_0^4 \frac{\pi^2 - 4.2}{8\pi^2 c_s^3} \left\langle \frac{1}{|\mathbf{k}-\mathbf{k}'|} \right\rangle \approx \frac{17.3(4\pi\varepsilon_0)^2}{4\pi^2 c_s^3 k_F}. \tag{32}$$

4 Superconductivity from Energy Fluctuations

4.1 General relations in 3D

For calculation of T_c we restrict ourselves to the densities sufficiently low such that $c_s k_F \gg E_F$ (leftmost and middle regime in Fig. 2 of the main text). In that case, the energy fluctuation attraction (21) reduces to an momentum-dependent instantaneous attractive potential. It can be further substituted with a constant value, averaging over momentum $\mathbf{q}^2 = (\mathbf{k}-\mathbf{k}')^2 = 2k_F^2(1-\cos\theta)$ from 0 to $4k_F^2$, assuming electrons close to the Fermi surface. To calculate T_c we use the formula [9] appropriate for weak instantaneous attraction, where the vertex corrections need to be treated due to the absence of a small parameter Ω_T/E_F:

$$T_c = \frac{e^\gamma E_F}{\pi}(2/e)^{7/3} e^{-1/\lambda}, \tag{33}$$

γ being the Euler's constant. The dimensionless coupling constant consists of the Coulomb and energy-fluctuation parts:

$$\lambda = \lambda_{En} + \lambda_{Coul}, \tag{34}$$

each one being equal to the corresponding interaction times the density of states $\nu_0 = \frac{m^*(3\pi^2 n)^{1/3}}{2\pi^2\hbar^2}$. The coupling constant due to the energy fluctuation exchange (neglecting LO phonon and cross-polarization contributions) is then

$$\begin{aligned} \lambda_{En} &= \frac{3(3\pi^2)^{1/3}}{8\pi^2}\left(\frac{g}{a_0^3}\right)^2 \frac{\varepsilon_0^2\Omega_0^4}{\Omega_T^3}\left(\frac{\hbar^2}{m^* a_0^2}\right)^{-1} a_0 n^{1/3}\left(\frac{1}{2} + \log\frac{\Omega_T}{\omega_T} - \overline{f}\right), \\ \overline{f} &= \frac{1}{2}\int_0^\pi \sin\theta d\theta f(\omega_q(\theta)/\omega_T) = \int_0^{2c_s k_F} \frac{dx}{2c_s^2 k_F^2} x f(x/\omega_T) \\ &\approx 1 (c_s k_F \ll \omega_T);\ \log\frac{2k_F c_s}{\omega_T} - \frac{1}{2}(c_s k_F \gg \omega_T). \end{aligned} \tag{35}$$

where in the prefactor $c_s q_D \to \Omega_T$, $q_D = (6\pi^2)^{1/3}/a_0$, a_0 being the lattice constant ($a_0^3 \approx v_c$ — the unit cell volume) is used. Due to the logarithmic dependence on the

upper cutoff, one has, in principle to use the full dispersion $\omega_T(\mathbf{q})$; here, we instead use an average c_s value in the spirit of the Debye approximation. On the other hand, as the transferred momentum is much smaller than q_D, we use the low-q value of c_s in $\overline{f}$.

The Coulomb repulsion (11) yields the dimensionless coupling:

$$\lambda_{Coul} = -\left(\frac{m^* p_F}{2\pi^2\hbar^3}\frac{e^2 c_s^2}{\varepsilon_0 \Omega_0^2} + \frac{(1+\log 2) m^* k_F}{2\pi^2\hbar^2}\frac{e^2\omega_T^2(n_e)}{2\varepsilon_0\Omega_0^2 k_F^2}\right). \tag{36}$$

4.2 Application to bulk doped $SrTiO_3$

The parameters used to calculate the $T_c(n_e)$ in the main text for the bulk doped $SrTiO_3$ are given in Table 1. For the densities, where bulk superconductivity occurs ($10^{18} - 10^{20}$ cm^{-3}) two bands are occupied [10]; however, since the mass of one of them is significantly larger $4m_e$ against $1.3m_e$ in the second band, we ignore the presence of the light band with smaller density of states. The maximal phonon energy Ω_T is around 22 meV [11, 12]. Note that the phonon dispersion is quite anharmonic: using the low-q value of sound velocity one obtains $c_s q_D \approx 40$ meV - larger than the actual Ω_T. Finally, we also neglect the effect of the tetragonal distortion that weakly splits the isotropic phonon into A_{2u} and E_{1u} modes [13]. Instead, the value of ω_T determined from ε has the meaning of an average energy of these modes.

ε	ω_T	Ω_0
$2 \cdot 10^4$ [14] [5]	$\frac{\Omega_0}{\sqrt{\varepsilon}} \approx 1.4$ meV	194.4 meV[15, 3]
c_s	m^*	Ω_T
$6.6 \cdot 10^5$cm/s [15, 3]	$4m_e$ [10]	22 meV [11, 12]

Table 1: Numerical values of relevant parameters for $SrTiO_3$

Let us now review the expressions above. The cross-polarization term (26), given that $c_s \approx \overline{c}_s/2$ is then small as 0.1 compared to the logarithm in the TO-phonon energy fluctuation contribution (21) and can be dropped. The LO contribution (25) is smaller then the TO one by $\left(1 + \frac{\Omega_0^2}{c_s^2\kappa_D^2}\right)^{-3/2} \sim 10^{-3}$ for densities around 10^{18} cm^{-3}. Consequently, both can be neglected and we take only the TO-phonon energy fluctuations into account. Evaluating the Coulomb repulsion terms (36) ($\varepsilon_0 = 1/4\pi$

used) we get:

$$\frac{m^* p_F}{2\pi^2\hbar^3}\frac{4\pi e^2 c_s^2}{\Omega_0^2} = \frac{2}{\pi}(3\pi^2)^{1/3}[n^{1/3}a_B]\frac{m^* c_s^2 \frac{e^2}{a_B}}{\Omega_0^2} \approx 0.74\cdot 10^{-8}(n[cm^{-3}])^{1/3}$$

$$\frac{(1+\log 2)m^* k_F}{2\pi^2\hbar^2}\frac{4\pi e^2 \omega_T^2(n_e)}{2\Omega_0^2 k_F^2} = \frac{(1+\log 2)}{\pi(3\pi^2)^{1/3}}\frac{m^*}{m}\frac{\omega_T^2(n_e)}{\Omega_0^2 a_B n^{1/3}} \approx 7\cdot 10^3\frac{\omega_T^2(n_e)}{\omega_T^2(0)}(n[cm^{-3}])^{-1/3}. \tag{37}$$

Combining (36) and (35) we get the dimensionless coupling constant used together with (33) to obtain the $T_c(n_e)$ in Fig. 3a of the main text. The value of the coupling constant $g/a_0^3 = 0.68$ has been obtained by fitting the experimental $T_c(n_e)$ data [16, 17]. The resulting values of the coupling constants are given in Fig. 3.

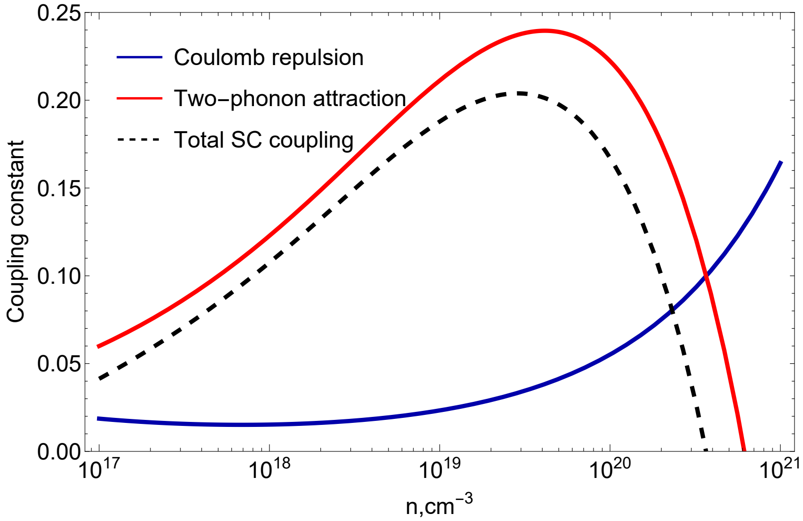

Figure 3: Magnitude of the screened Coulomb repulsion (blue) (36), energy-fluctuation attraction (red) (35) and the total interaction λ in the pairing channel (black dashed) as a function of density for $g/a_0^3 = 0.68$. TO phonon self-energy due to the energy coupling 8 is taken into account.

4.3 2D layers

For the 2D layer, we have assumed a two-unit-cell thick STO layer ($l_0 = 2a_0$) and $m^* = 1.8m_e$, appropriate for the lowest densities in $SrTiO_3$ [10], as the relevant 3D

electron densities in this case go down to 10^{17} cm^{-3}. The dimensionless coupling constant is taken equal to 0.68, as in the 3D case, and we also neglected the effect of tetragonal anisotropy on the phonon energy. T_c for 2D case obtained using the same approach as in 3D [9] is given by [18]

$$T_c^{2D} \approx 0.15 E_F e^{-1/\lambda^{2D}}, \tag{38}$$

where (using the 2D density of states $\nu_0^{2D} = \frac{m^*}{2\pi\hbar^2}$) :

$$\lambda^{2D} = \lambda_{En}^{2D} + \lambda_{Coul}^{2D},$$

$$\lambda_{En}^{2D} = \left(\frac{g}{a_0^3}\right)^2 \left(\frac{l}{a_0}\right)^{-2} \frac{4\varepsilon_0^2\Omega_0^4}{8\pi^2(\hbar c_s/a_0)^2} \left(\frac{\hbar^2}{m^* a_0^2}\right)^{-1} \left(\left\langle \frac{\arctan \frac{\omega_q}{2\omega_T}}{\omega_q} \right\rangle_{0<\varphi<2\pi}^{q^2=4k_F^2 \sin^2 \frac{\varphi}{2}} - \frac{1}{\pi^2 c_s k_F} I(\omega_T/c_s k_F) \right),$$

$$I(z) = \int_0^\infty dx \int_0^\pi d\theta \int_0^{2\pi} d\varphi \frac{4x^3\left(1-\cos^2\theta\right)}{\sqrt{\sin^2\left(\frac{\varphi}{2}\right)\left(\left(x^2+1\right)^2 - 4x^2\cos^2\theta\right)} \sqrt{\left(z^2\csc^2\left(\frac{\varphi}{2}\right) + x^2 + 1\right)^2 - 4x^2\cos^2\theta}} \frac{1}{\left(\sqrt{-2x\cos\theta + z^2\csc^2\left(\frac{\varphi}{2}\right) + x^2 + 1} + \sqrt{2x\cos\theta + z^2\csc^2\left(\frac{\varphi}{2}\right) + x^2 + 1}\right)},$$

$$\lambda_{Coul}^{2D} = \left\langle \frac{(4\pi\varepsilon_0)^{-1}}{2 + \frac{\hbar^2}{2m^* e^2} \frac{\Omega_0^2 l_0 q^2}{\omega_n^2 + \omega_T^2(n_e) + c_s^2 q^2}} \right\rangle_{0<\varphi<2\pi}^{q^2=4k_F^2 \sin^2 \frac{\varphi}{2}}. \tag{39}$$

Note that the contribution of cross-polarization TO-LO term had to be included into the energy fluctuation coupling. Unlike the 3D case, integrals do not diverge at large momenta and hence we always use the low-momentum value of c_s. Using the parameters for $SrTiO_3$ shown above, one gets the results of Fig. 3b of the main text.

References

[1] H. Kleinert and Adriaan M. J. Schakel. Gauge-Invariant Critical Exponents for the Ginzburg-Landau Model. *Phys. Rev. Lett.*, 90:097001, Mar 2003.

[2] Rahul Nandkishore, Max A. Metlitski, and T. Senthil. Orthogonal metals: The simplest non-fermi liquids. *Phys. Rev. B*, 86:045128, Jul 2012.

[3] Abhishek Kumar, Vladimir I. Yudson, and Dmitrii L. Maslov. Quasiparticle and nonquasiparticle transport in doped quantum paraelectrics. *Phys. Rev. Lett.*, 126:076601, Feb 2021.

[4] Jonathan Ruhman and Patrick A. Lee. Superconductivity at very low density: The case of strontium titanate. *Phys. Rev. B*, 94:224515, Dec 2016.

[5] Clement Collignon, Xiao Lin, Carl Willem Rischau, Benoit Fauque, and Kamran Behnia. Metallicity and superconductivity in doped strontium titanate. *Annual Review of Condensed Matter Physics*, 10(1):25–44, 2019.

[6] LV Keldysh. Coulomb interaction in thin semiconductor and semimetal films. *JETP Letters*, 29:658, 1979.

[7] Pierluigi Cudazzo, Ilya V. Tokatly, and Angel Rubio. Dielectric screening in two-dimensional insulators: Implications for excitonic and impurity states in graphane. *Phys. Rev. B*, 84:085406, Aug 2011.

[8] Thibault Sohier, Marco Gibertini, Matteo Calandra, Francesco Mauri, and Nicola Marzari. Breakdown of optical phonons' splitting in two-dimensional materials. *Nano Letters*, 17(6):3758–3763, 2017. PMID: 28517939.

[9] L. P. Gor'kov and T. K. Melik-Barkhudarov. Contribution to the theory of superconductivity in an imperfect fermi gas. *Soviet Physics JETP*, 13(5), 1961.

[10] Xiao Lin, German Bridoux, Adrien Gourgout, Gabriel Seyfarth, Steffen Krämer, Marc Nardone, Benoît Fauqué, and Kamran Behnia. Critical doping for the onset of a two-band superconducting ground state in $\mathrm{srtio}_{3-\delta}$. *Phys. Rev. Lett.*, 112:207002, May 2014.

[11] W G Stirling. Neutron inelastic scattering study of the lattice dynamics of strontium titanate: harmonic models. *Journal of Physics C: Solid State Physics*, 5(19):2711–2730, oct 1972.

[12] D. Bäuerle, D. Wagner, M. Wöhlecke, B. Dorner, and H. Kraxenberger. Soft modes in semiconducting srtio3: Ii. the ferroelectric mode. *Zeitschrift für Physik B Condensed Matter*, 38(4):335–339, Dec 1980.

[13] A Yamanaka, M Kataoka, Y Inaba, K Inoue, B Hehlen, and E Courtens. Evidence for competing orderings in strontium titanate from hyper-raman scattering spectroscopy. *Europhysics Letters (EPL)*, 50(5):688–694, jun 2000.

[14] Maria N. Gastiasoro, Jonathan Ruhman, and Rafael M. Fernandes. Superconductivity in dilute $SrTiO_3$: A review. *Annals of Physics*, 417:168107, 2020.

[15] Yasusada Yamada and Gen Shirane. Neutron scattering and nature of the soft optical phonon in srtio3. *Journal of the Physical Society of Japan*, 26(2):396–403, 1969.

[16] C. S. Koonce, Marvin L. Cohen, J. F. Schooley, W. R. Hosler, and E. R. Pfeiffer. Superconducting Transition Temperatures of Semiconducting $SrTiO_3$. *Phys. Rev.*, 163:380–390, Nov 1967.

[17] Clément Collignon, Benoît Fauqué, Antonella Cavanna, Ulf Gennser, Dominique Mailly, and Kamran Behnia. Superfluid density and carrier concentration across a superconducting dome: The case of strontium titanate. *Phys. Rev. B*, 96:224506, Dec 2017.

[18] Lev P. Gor'kov. Superconducting transition temperature: Interacting fermi gas and phonon mechanisms in the nonadiabatic regime. *Phys. Rev. B*, 93:054517, Feb 2016.
